# Prevalence of Fluoroquinolone-Resistant *Campylobacter* Species in Iran: A Systematic Review and Meta-Analysis

**DOI:** 10.1155/2020/8868197

**Published:** 2020-10-30

**Authors:** Farzad Khademi, Amirhossein Sahebkar

**Affiliations:** ^1^Department of Microbiology, School of Medicine, Ardabil University of Medical Sciences, Ardabil, Iran; ^2^Neurogenic Inflammation Research Center, Mashhad University of Medical Sciences, Mashhad, Iran; ^3^Biotechnology Research Center, Pharmaceutical Technology Institute, Mashhad University of Medical Sciences, Mashhad, Iran; ^4^Halal Research Center of IRI, FDA, Tehran, Iran

## Abstract

**Background:**

*Campylobacter* species are one of the main causes of bacterial food poisoning worldwide. Recently, WHO reported that the emergence of fluoroquinolone-resistant *Campylobacter* species is becoming a public health issue around the world. The aim of the present systematic review and meta-analysis was to evaluate the prevalence of the antimicrobial susceptibility patterns of *Campylobacter* species, especially fluoroquinolone-resistant strains isolated from human and animal origins in Iran.

**Methods:**

Using related keywords and without date and language limitations, a comprehensive literature search was conducted in PubMed, Scopus, ISI Web of Knowledge, Google Scholar, and SID to identify relevant studies on the prevalence of the antimicrobial susceptibility patterns of *Campylobacter* species in Iran.

**Results:**

A total of 34 reports (9 in Persian and 25 in English) were selected based on inclusion and exclusion criteria. Disk diffusion, *E*-test, and agar dilution were common methods used for antimicrobial susceptibility testing. The antibiotic resistance profiles of *Campylobacter* species against fluoroquinolones were as follows: 53.6%, 41.8%, and 0% to ciprofloxacin for *C. jejuni*, *C. coli*, and *C. lari*, respectively, 24.3% and 25.1% to enrofloxacin for *C. jejuni* and *C. coli*, respectively, 59.6% and 49.2% to nalidixic acid for *C. jejuni* and *C. coli*, respectively, and 87.3% and 64.7% to ofloxacin for *C. jejuni* and *C. coli*, respectively.

**Conclusion:**

Our findings revealed a high prevalence of fluoroquinolone-resistant *Campylobacter* species in Iran. This calls for the use of more effective antibiotics with low resistance rates including aminoglycosides, chloramphenicol, and imipenem.

## 1. Introduction

The genus *Campylobacter* includes small, motile, and curved Gram-negative bacteria [1–3]. These rod-shaped bacteria are thermophilic, 30°C to 46°C, and microaerophilic, 5% O_2_, and belong to the family Campylobacteraceae [4]. *Campylobacter* is a zoonotic pathogen that is colonized in the intestinal tract of domestic and wild animals and birds and can infect human through consumption of contaminated water, different foods such as raw or uncooked meat, unpasteurized milk, and contact with infected animals or (rarely) human [4, 5]. Poultry, cattle, sheep, pigs, birds, dogs, and cats are common reservoir hosts for human infections [2]. This food-borne bacterial pathogen is the major cause of bacterial gastroenteritis and septicemia in humans in both developing and developed countries [1, 2]. In developed countries, *Campylobacter* bacteria are the most important causative agents for gastrointestinal infection [6]. It is estimated that between 400 and 500 million individuals become infected with *Campylobacter* species in the world annually [4, 5]. The most common species associated with bacterial gastroenteritis in human are *Campylobacter jejuni* and *Campylobacter coli* as well as *Campylobacter fetus* associated with systemic infections [2, 4]. Additionally, in some cases, these enteric pathogens are associated with two immune-related late complications, i.e., Guillain-Barré syndrome and reactive arthritis [1, 2]. The severity of *Campylobacter* infections varies from a mild and self-limiting illness to severe infections [5]. For the treatment of self-limiting intestinal infections, the replacement of fluids and electrolytes is enough, while severe extraintestinal *Campylobacter* infections such as septicemia, endocarditis, and septic thrombophlebitis should be treated with appropriate antibiotics [2, 7]. Macrolides, including erythromycin and azithromycin, and fluoroquinolones are considered as the drugs of choice for infected patients [2, 4, 5]. Alternative treatments are tetracyclines and gentamicin [2, 4, 5]. Additionally, *Campylobacter* strains are susceptible to amoxicillin/clavulanic acid, imipenem, aminoglycosides, chloramphenicol, and clindamycin [2]. However, in recent years, antimicrobial resistance of *Campylobacter* species has been increased in both developing and developed countries and is becoming a global problem [8]. It is noteworthy that resistance to penicillins, cephalosporins, and sulfonamides has emerged [2]. On the other hand, in February 2017, the World Health Organization (WHO) announced that fluoroquinolone-resistant *Campylobacter* species are growing globally, calling for a priority to find effective antibiotics [9]. Therefore, the aim of the present systematic review and meta-analysis was to evaluate the antimicrobial susceptibility patterns of *Campylobacter* species, especially fluoroquinolone-resistant strains, isolated from human and animal origins in Iran.

## 2. Methods

### 2.1. Literature Search and Selection Criteria

We started at 1 August 2018 for a comprehensive literature search in international search engines including PubMed (https://www.ncbi.nlm.nih.gov), Scopus (https://www.scopus.com), ISI Web of Knowledge (https://www.isiwebofknowledge.com), Google Scholar (http://scholar.google.com), and Scientific Information Database (SID) (http://www.sid.ir), a national database, on each report about the prevalence of the antimicrobial susceptibility patterns of *Campylobacter* species in Iran. There was no date and language limitation for searching, and related keywords used were antibiotic resistance, *Campylobacter* species (*C. jejuni*, *C. coli*, and *C. lari*), and Iran. A number of missed studies were obtained by reviewing the list of references and searching for journals. The meta-analysis was performed step by step based on the PRISMA recommendations [10].

Inclusion or exclusion criteria for assessing eligibility in the study were all types of Persian- and English-language articles which had enough data on the prevalence of the resistance patterns of campylobacters, in species level, to different antibiotics in Iran. Review articles, case reports, abstracts of articles, and duplicates were excluded. Articles evaluating the resistance patterns of campylobacters only at the genus level or the resistance genes and those studies with unclear results were excluded.

### 2.2. Data Extraction of Articles

After completely reviewing all included studies by two authors, needed information was extracted and placed in Tables 1–3 based on organism species type. The quality of data was evaluated based on the Newcastle-Ottawa scale adapted for cross-sectional studies (data has not been shown). The checklist of items was based on three criteria including selection (representativeness of the sample, sample size, nonrespondents, and ascertainment of the exposure) (maximum 5 stars), comparability (comparability of outcome groups) (maximum 2 stars), and outcome (assessment of the outcome and statistical test) (maximum 3 stars).

Data obtained from eligible studies include publishing year, location of the study, number of strains, origin of samples, methods used for antimicrobial susceptibility testing, and antibiotic resistance profiles of *C. jejuni*, *C. coli*, and *C. lari*.

### 2.3. Meta-Analysis

The data for the quantitative data synthesis were transferred to the Comprehensive Meta-Analysis (CMA) software (Biostat, Englewood, NJ). Resistance rates of *C. jejuni*, *C. coli*, and *C. lari* were calculated for each antibiotic as a percentage and expressed as 95% confidence intervals (95% CIs). *I*^2^ statistic was used to evaluate the existed heterogeneity, and considering the percent of inconsistency among studies, pooling of data was performed using fixed-or random-effects models. The assessment of publication bias was done using funnel plots.

## 3. Results

### 3.1. Characteristics of Included Studies

**Figure 1:**
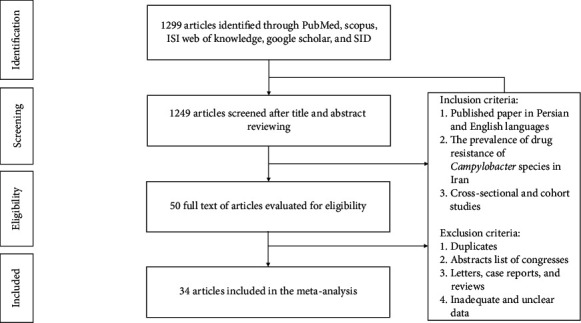
A summary of the study selection processes.

As shown in Figure 1, a total of 1299 articles were obtained from five databases (PubMed, Scopus, ISI Web of Knowledge, Google Scholar, and SID). According to the presented inclusion or exclusion criteria in Figure 1, 1249 articles were removed and the eligibility of 50 remained articles was evaluated. Among them, 16 studies did not meet inclusion criteria because of reporting the resistance patterns of campylobacters only in the genus level or had inadequate information, while 34 articles (9 in Persian and 25 in English) had complete data and were included in our meta-analysis.

**Figure 2:**
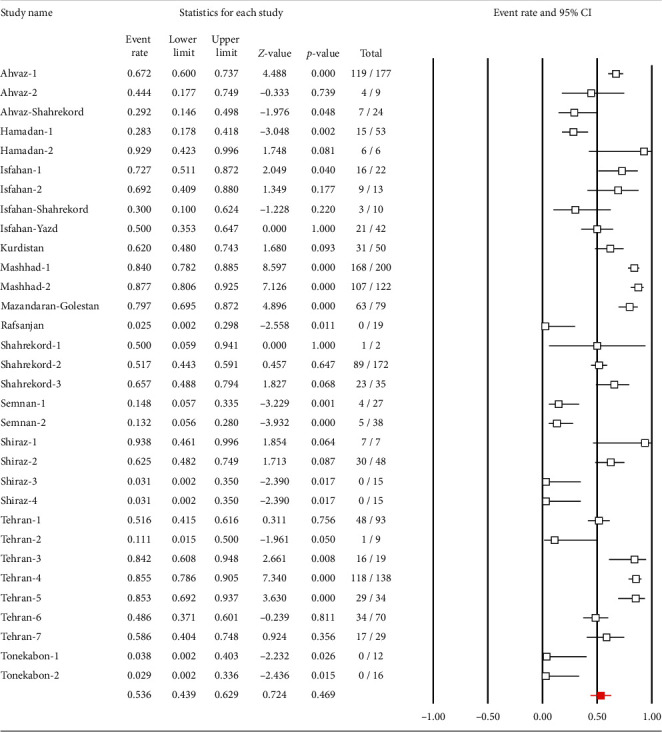
Funnel plot of the meta-analysis of the prevalence of *C. jejuni* resistant to ciprofloxacin in Iran.

**Figure 3:**
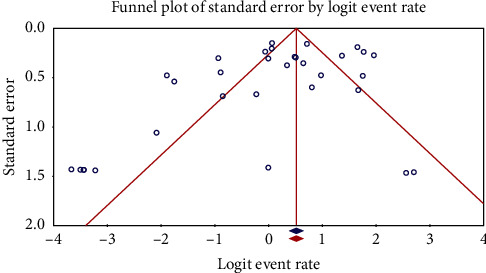
Funnel plot of the meta-analysis of the prevalence of *C. coli* resistant to ciprofloxacin in Iran.

The characteristics of the 34 included studies are summarized in Tables 1–3. The main data was extracted from 3 studies from Ahvaz [11–13], 2 studies from Hamadan [14, 15], 4 studies from Isfahan [16–19], 1 study from Kerman [20], 1 study from Kurdistan [21], 2 studies from Mashhad [22, 23], 1 study from Mazandaran and Golestan [24], 1 study from Rafsanjan [25], 5 studies from Shahrekord [13, 18, 26–28], 2 studies from Semnan [29, 30], 4 studies from Shiraz [31–34], 8 studies from Tehran [35–42], 2 studies from Tonekabon [34, 43], 1 study from Yazd [19], and 1 study from Zahedan [44]. Disk diffusion, *E*-test, and agar dilution were the most common methods used to evaluate antibiotic-resistant *Campylobacter* species in Iran (Tables 1–3). Additionally, the most common *Campylobacter* species for which their antibiotic resistance has been evaluated were *C. jejuni*, *C. coli*, and *C. lari*. The origins of *Campylobacter* species were human and animal fecal samples as well as food samples with animal origin including milk, dairy products, and animal meats like poultry, cattle, sheep, camels, beef, water buffalo, ducks, and geese. A random-effects model was used for pooling data on the prevalence of antibiotic resistance of *Campylobacter* species due to the presence of high heterogeneity (*I*^2^ > 25%). There was some evidence of publication bias (Figures 2 and 3).

### 3.2. Characteristics of *C. jejuni* Antibiotic Resistance

**Figure 4:**
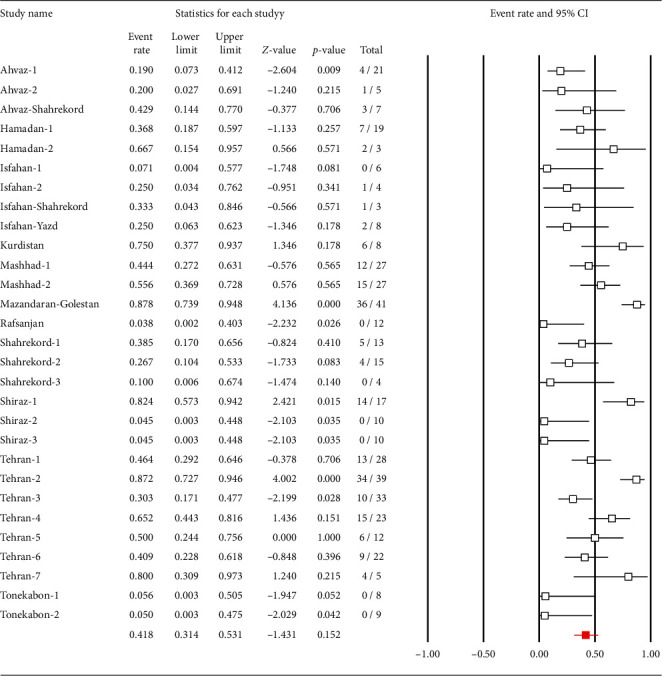
Forest plot of the meta-analysis of the prevalence of *C. jejuni* resistant to ciprofloxacin in Iran.

In our presented meta-analysis, a total of 34 studies determined the prevalence of *C. jejuni* antibiotic resistance (Table 1). Antimicrobial resistance patterns of *C. jejuni* in Iran were as follows: 22.8% (95% CI: 15.9–31.6) to ampicillin, 17.7% (95% CI: 11.4–26.5) to amoxicillin, 25.8% (95% CI: 14.5–41.7) to colistin, 24.3% (95% CI: 16.8–33.7) to enrofloxacin, 2.3% (95% CI: 0.8–6.4) to spectinomycin, 8.6% (95% CI: 3.9–17.7) to neomycin, 7.1% (95% CI: 4.7–10.7) to streptomycin, 6% (95% CI: 4.1–8.6) to chloramphenicol, 50.7% (95% CI: 41.1–60.4) to tetracycline, 66.9% (95% CI: 40.5–85.8) to trimethoprim/sulfamethoxazole, 41.2% (95% CI: 25.1–59.5) to cefotaxime, 6.4% (95% CI: 3.6–11.1) to erythromycin, 53.6% (95% CI: 43.9–62.9) to ciprofloxacin (Figure 4), 0% to imipenem, 4.5% (95% CI: 2.5–7.7) to gentamicin, 9.5% (95% CI: 0.6–65.5) to meropenem, 89.4% (95% CI: 73.8–96.2) to cephalothin, 59.6% (95% CI: 52.1–66.7) to nalidixic acid, 54.6% (95% CI: 38.9–69.4) to ceftazidime and 76.5% (95% CI: 54.5–89.8) to cephalexin. Additionally, other antibiotic resistance patterns were as follows: clindamycin 4 (66.6%), tylosin 15 (31.2%), oxacillin 9 (100%), amikacin 4 (5.1%), azithromycin 0 (0%), ceftriaxone 9 (56.2%), amoxi-clave 16 (100%), penicillin 16 (100%), vancomycin 16 (100%), tobramycin 4 (25%), ofloxacin 55 (87.3%), and carbenicillin 25 (39.6%).

### 3.3. Characteristics of *C. coli* Antibiotic Resistance

**Figure 5:**
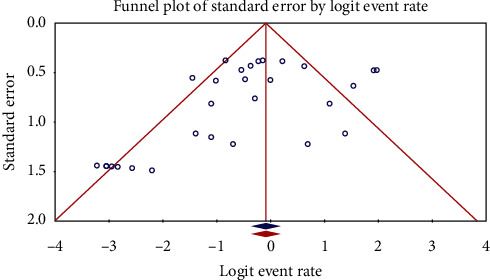
Forest plot of the meta-analysis of the prevalence of *C. coli* resistant to ciprofloxacin in Iran.

The characteristics of the 29 studies that determined the prevalence of *C. coli* antibiotic resistance are summarized in Table 2. The prevalence of resistance of *C. coli* to different antibiotics was as follows: 24.5% (95% CI: 14.5–38.4) to ampicillin, 23.5% (95% CI: 13.7–37.2) to amoxicillin, 23.1% (95% CI: 12.1–39.5) to colistin, 25.1% (95% CI: 19.2–32.1) to enrofloxacin, 5.4% (95% CI: 2–13.5) to spectinomycin, 8.3% (95% CI: 4.7–14.1) to neomycin, 11.6% (95% CI: 5.4–23.3) to streptomycin, 9.6% (95% CI: 4.9–17.8) to chloramphenicol, 47.7% (95% CI: 35.6–60.1) to tetracycline, 67.2% (95% CI: 33.6–89.3) to trimethoprim/sulfamethoxazole, 51.5% (95% CI: 35.8–66.9) to cefotaxime, 13% (95% CI: 6.9–23) to erythromycin, 41.8% (95% CI: 31.4–53.1) to ciprofloxacin (Figure 5), 0% to imipenem, 6.8% (95% CI: 4.3–10.5) to gentamicin, 27.2% (95% CI: 1.2–92.2) to meropenem, 65.5% (95% CI: 50.1–78.2) to cephalothin, 49.2% (95% CI: 36.6–61.9) to nalidixic acid, 62.2% (95% CI: 31.8–85.2) to ceftazidime, and 73% (95% CI: 38.6–92.1) to cephalexin. Additionally, other antibiotic resistance patterns were as follows: clindamycin 2 (66.6%), tylosin 2 (11.7%), oxacillin 5 (100%), amikacin 3 (7.3%), ceftriaxone 9 (100%), amoxi-clave 9 (100%), penicillin 9 (100%), vancomycin 9 (100%), tobramycin 0 (0%), ofloxacin 11 (64.7%), and carbenicillin 7 (41.1%).

### 3.4. Characteristics of *C. lari* Antibiotic Resistance

A total of 4 studies investigating the prevalence of *C. lari* antibiotic resistance were included in the meta-analysis (Table 3). Antimicrobial resistance patterns of *C. lari* in Iran were as follows: 60% (95% CI: 19–90.5) to ampicillin, 93.7% (95% CI: 46.1–99.6) to amoxicillin, 14.3% (95% CI: 2–58.1) to streptomycin, 20.9% (95% CI: 10.8–36.7) to chloramphenicol, 10.5% (95% CI: 4–24.9) to tetracycline, 16.7% (95% CI: 4.2–47.7) to trimethoprim/sulfamethoxazole, 70.4% (95% CI: 51.3–84.3) to cefotaxime, 7.4% (95% CI: 2.4–20.6) to erythromycin, 0% to ciprofloxacin, 12.7% (95% CI: 5.1–28.5) to gentamicin, and 63.2% (95% CI: 32.7–85.9) to cephalexin. Additionally, other antibiotic resistance patterns were as follows: ceftriaxone 7 (100%), amikacin 0 (0%), amoxi-clave 7 (100%), penicillin 7 (100%), vancomycin 7 (100%) and tobramycin 2 (29%).

Abbreviations: AMP: ampicillin; AMX: amoxicillin; CST: colistin; NFX: enrofloxacin; SPT: spectinomycin; NEO: neomycin; STR: streptomycin; CHL: chloramphenicol; TET: tetracycline; TMP/SMX: trimethoprim/sulfamethoxazole; CTX: cefotaxime; ERY: erythromycin; CIP: ciprofloxacin; IPM: imipenem; GEN: gentamicin; MEM: meropenem; CEF: cephalothin; NAL: nalidixic acid; CAZ: ceftazidime; LEX: cephalexin; AST: antimicrobial susceptibility testing; ND: not determined.

## 4. Discussion

Food-borne illnesses caused by *Campylobacter* species as well as other bacteria related to food poisoning can be prevented by avoiding food contamination and growth of bacteria through proper food preparation and proper cooking as well as avoidance of contamination of water sources and consuming pasteurized dairy products [2, 45]. However, the main problem is food contamination with drug-resistant pathogens, which is a major threat to public health [46]. Antibiotic resistance genes can be transferred among food-borne pathogens, and this makes the treatment of severe infections difficult [46]. Today, fluoroquinolone-resistant *Campylobacter* species have turned into a global concern [9]. Fluoroquinolones are selective drugs in the treatment of campylobacteriosis; however, an increasing trend of resistance in *Campylobacter* species isolated from human and animal origins has been reported in the USA and Canada (19–47%), European countries (17–99%), and Africa and Asia (>80%) [5]. According to the present study, the resistance of *Campylobacter* species isolated from human and animal origins to quinolones and fluoroquinolones including ciprofloxacin, nalidixic acid, enrofloxacin, and ofloxacin was also prevalent in Iran and varied from 0% to 87.3% (Tables 1–3). Efflux pumps, CmeABC, and single point mutations in DNA gyrase A (GyrA) such as C257 T mutation, the most frequent mutation, are involved in chromosomally mediated quinolone resistance in *Campylobacter* species [5].

Macrolides are also recommended as another selective antibiotic class for the treatment of campylobacteriosis [2, 5]. The resistance rate to erythromycin in *Campylobacter* species isolated from human and animal samples in Iran was low (6.4%, 13%, and 7.4% for *C. jejuni*, *C. coli*, and *C. lari*, respectively). The frequency of erythromycin resistance in Iran was higher than that in Turkey [47], Ethiopia [48], Canada [49], Australia [50], and the Czech Republic [51] and lower than that of South Africa [52], Malaysia [53], Italy [54], and China [55]. The target modifications through point mutations in the *23S rRNA* gene such as A2074C, A2074G, and A2075G mutations, modifying L4 and L22 ribosomal proteins along with CmeABC efflux pump, are three main mechanisms complicated in macrolide resistance in *Campylobacter* species [5].

Tetracyclines and gentamicin have also importance in *Campylobacter* infection therapy as second-line antibiotics [2, 4, 5]. The *tet*(*O*) gene, which encodes a ribosomal protection protein, and CmeABC multidrug efflux pump are associated with tetracycline resistance in *Campylobacter* species [5]. Additionally, aminoglycoside-modifying enzymes play an important role in aminoglycoside resistance in *Campylobacter* species [5]. Based on the results of this study, *C. jejuni* and *C. coli* antibiotic resistance rates to tetracycline were much higher than gentamicin. Similar results were observed in the studies reported from Turkey [47], Italy [54], South Korea [56], and Poland [57]. Moreover, similar to our results, high rates of tetracycline resistance were reported in the studies of Turkey [47], Canada [49], South Africa [52], Malaysia [53], Italy [54], China [55], South Korea [56], and Poland [57]. Noteworthy, the determination of *Campylobacter* species susceptibility patterns against other antibiotics has received less attention. One reason could be attributed to the high sensitivity of bacteria to these antibiotics. For example, in the current study, antibiotic resistance pattern to protein synthesis inhibitors was low. On the other hand, resistance to protein synthesis inhibitors was lower than cell growth inhibitors and folic acid metabolism inhibitors.

As shown in other studies, the efficacy of cell growth inhibitor antibiotics against *Campylobacter* species is limited [5]. Our study also showed that among the three evaluated *Campylobacter* species, antibiotic resistance to *β*-lactam antibiotics, especially penicillins and cephalosporins, was high. On the other hand, the resistance rate to imipenem was lower than meropenem. The intrinsic resistance and *β*-lactamase enzymes are two main mechanisms of resistance to *β*-lactam antibiotics in *Campylobacter* species [5]. The intrinsic resistance is also the main resistance mechanism of *Campylobacter* species against vancomycin and folic acid metabolism inhibitors [5]. Our results confirmed high resistance rates to these antibiotics probably due to intrinsic resistance.

## 5. Conclusion

In accordance with the WHO report on fluoroquinolone-resistant *Campylobacter* species in the world and the urgent need to develop new antibiotics, our meta-analysis showed a high prevalence of resistance of *Campylobacter* species isolated from human and animal origins to quinolones and fluoroquinolones in Iran. On the other hand, compared to penicillins, cephalosporins, and sulfonamides, *Campylobacter* species were susceptible or showed low resistance rates to aminoglycosides, chloramphenicol, and imipenem. Therefore, these antibiotics could be recommended for the treatment of campylobacteriosis in Iran. We recommend monitoring antibiotic-resistant *Campylobacter* species through continuous drug sensitivity monitoring and investigating resistance mechanisms, especially against fluoroquinolones, to prevent further expansion of resistant species in Iran.

## Figures and Tables

**Table 1:** The antibiotic resistance profiles of *C. jejuni*.

Year	City	Strains (*n*)	Origin	AST	Antibiotic resistance (*n*)																			
					AMP	AMX	CST	NFX	SPT	NEO	STR	CHL	TET	TMP-SMX	CTX	ERY	CIP	IPM	GEN	MEM	CEF	NAL	CAZ	LEX
2007-2008	Ahvaz	177	Animal	Disk diffusion	33	14	ND	85	ND	ND	11	1	141	ND	ND	3	119	ND	0	ND	ND	105	ND	ND
2007-2008	Ahvaz	9	Human	Disk diffusion E-test	9	ND	ND	ND	ND	ND	ND	ND	6	ND	6	5	4	ND	0	ND	9	7	6	ND
2009-2010	Ahvaz-Shahrekord	24	Animal	Disk diffusion	4	1	ND	6	ND	ND	2	2	18	ND	ND	0	7	ND	1	ND	ND	9	ND	ND
2016	Hamadan	53	Animal	Disk diffusion	23	22	43	ND	ND	ND	11	3	10	44	ND	3	15	ND	0	ND	40	0	ND	ND
2013-2014	Hamadan	6	Human	Disk diffusion	ND	ND	ND	ND	ND	ND	ND	1	5	ND	ND	4	6	ND	3	3	ND	2	ND	ND
2014-2015	Isfahan	22	Animal	Disk diffusion	1	4	ND	5	ND	ND	2	1	18	ND	ND	1	16	ND	0	ND	ND	12	ND	ND
2006–2008	Isfahan	13	Animal	Disk diffusion	1	3	ND	3	ND	ND	1	0	5	ND	ND	0	9	ND	0	ND	ND	7	ND	ND
2011-2012	Isfahan-Shahrekord	10	Animal	Disk diffusion	1	0	ND	0	ND	ND	1	0	4	ND	ND	0	3	ND	0	ND	ND	5	ND	ND
2008-2009	Isfahan-Yazd	42	Animal	Disk diffusion	5	1	ND	10	ND	ND	3	0	28	ND	ND	0	21	ND	0	ND	ND	18	ND	ND
2007–2009	Kerman	190	Animal	Disk diffusion	103	ND	ND	ND	ND	ND	ND	ND	103	173	ND	ND	ND	ND	ND	ND	ND	ND	ND	ND
2015-2016	Kurdistan	50	Animal	Disk diffusion agar dilution	5	10	ND	ND	ND	ND	ND	ND	34	ND	ND	1	31	ND	0	0	ND	ND	ND	ND
2013	Mashhad	200	Animal	Disk diffusion	13	26	ND	60	ND	42	ND	9	181	ND	ND	76	168	ND	7	ND	ND	132	ND	ND
2012	Mashhad	122	Animal	Disk diffusion	20	4	25	11	1	8	6	6	88	ND	ND	1	107	ND	0	ND	ND	91	ND	ND
2014-2015	Mazandaran-Golestan	79	Animal	Disk diffusion	26	40	ND	28	ND	ND	15	ND	59	ND	ND	5	63	ND	1	ND	ND	59	ND	ND
2010	Rafsanjan	19	Animal	Disk diffusion	ND	ND	ND	ND	ND	ND	ND	ND	ND	19	ND	0	0	ND	19	ND	ND	0	ND	ND
2014	Shahrekord	2	Animal	Disk diffusion	1	1	ND	1	ND	ND	1	0	0	ND	ND	0	1	ND	0	ND	ND	1	ND	ND
2009-2010	Shahrekord	172	Animal	Disk diffusion	29	3	ND	20	ND	ND	3	4	125	ND	ND	2	89	ND	0	ND	ND	96	ND	ND
ND	Shahrekord	35	Animal	Disk diffusion	2	6	ND	14	ND	ND	3	1	28	ND	ND	1	23	ND	0	ND	ND	18	ND	ND
2007	Semnan	27	Human	Disk diffusion	ND	ND	ND	ND	ND	ND	ND	ND	9	12	ND	2	4	ND	1	ND	ND	ND	ND	ND
2007	Semnan	38	Human	Disk diffusion	ND	ND	ND	ND	ND	ND	ND	ND	11	20	ND	2	5	ND	1	ND	ND	ND	ND	ND
2014-2015	Shiraz	7	Human	Disk diffusion	5	ND	ND	ND	ND	ND	ND	ND	7	ND	ND	ND	7	ND	0	0	ND	7	ND	ND
2011–2013	Shiraz	48	Animal	Disk diffusion	22	ND	0	8	ND	0	ND	4	13	ND	13	3	30	ND	4	ND	44	7	ND	ND
ND	Shiraz	15	Animal	Disk diffusion E-test	2	ND	ND	ND	ND	ND	ND	4	1	2	4	1	0	ND	2	ND	ND	ND	ND	8
ND	Shiraz	15	Animal	Disk diffusion E-test	2	ND	ND	ND	ND	ND	ND	4	1	ND	7	1	0	ND	2	ND	ND	ND	ND	8
2011-2012	Tehran	93	Animal	Disk diffusion	12	26	32	ND	4	8	5	4	33	ND	ND	4	48	ND	3	ND	ND	66	ND	ND
2011	Tehran	9	Human	Disk diffusion	ND	ND	ND	ND	ND	ND	ND	ND	3	5	ND	2	1	ND	0	ND	ND	ND	ND	ND
2010	Tehran	19	Animal	Disk diffusion	19	ND	ND	ND	ND	11	ND	ND	5	ND	ND	ND	16	ND	ND	ND	ND	18	ND	ND
2008–2010	Tehran	138	Animal	Disk diffusion	15	44	32	ND	ND	3	3	6	109	ND	ND	5	118	ND	0	ND	ND	103	ND	ND
2008-2009	Tehran	34	Human	Disk diffusion	6	ND	5	ND	ND	4	3	3	12	ND	19	4	29	0	2	ND	ND	27	21	ND
2006-2007	Tehran	70	Animal	Disk diffusion	5	18	22	ND	1	2	2	1	22	ND	ND	1	34	ND	0	ND	ND	47	ND	ND
2004-2005	Tehran	29	Human	Disk diffusion	3	ND	0	ND	ND	1	0	1	7	ND	3	1	17	0	0	ND	28	22	12	27
ND	Tonekabon	12	Animal	Disk diffusion E-test	12	ND	ND	ND	ND	ND	ND	2	2	ND	9	3	0	ND	4	ND	ND	ND	ND	10
ND	Tonekabon	16	Animal	Disk diffusion	16	16	ND	ND	ND	ND	1	1	1	ND	ND	2	0	ND	1	ND	ND	ND	ND	14
2011–2013	Zahedan	19	Human	Disk diffusion	ND	ND	ND	ND	ND	ND	ND	ND	ND	ND	ND	0	ND	ND	ND	ND	ND	ND	ND	ND

Abbreviations: AMP: ampicillin; AMX: amoxicillin; CST: colistin; NFX: enrofloxacin; SPT: spectinomycin; NEO: neomycin; STR: streptomycin; CHL: chloramphenicol; TET: tetracycline; TMP/SMX: trimethoprim/sulfamethoxazole; CTX: cefotaxime; ERY: erythromycin\; CIP: ciprofloxacin; IPM: imipenem; GEN: gentamicin; MEM: meropenem; CEF: cephalothin; NAL: nalidixic acid; CAZ: ceftazidime; LEX: cephalexin; AST: antimicrobial susceptibility testing; ND: not determined.

**Table 2:** Antibiotic resistance profiles of *C. coli*.

Year	City	Strains (*n*)	Origin	AST	Antibiotic resistance (*n*)																			
					AMP	AMX	CST	NFX	SPT	NEO	STR	CHL	TET	TMP-SMX	CTX	ERY	CIP	IPM	GEN	MEM	CEF	NAL	CAZ	LEX
2007-2008	Ahvaz	21	Animal	Disk diffusion	4	0	ND	3	ND	ND	1	0	14	ND	ND	0	4	ND	0	ND	ND	11	ND	ND
2007-2008	Ahvaz	5	Human	Disk diffusion *E*-test	5	ND	ND	ND	ND	ND	ND	ND	3	ND	3	4	1	ND	1	ND	5	2	4	ND
2009-2010	Ahvaz-Shahrekord	7	Animal	Disk diffusion	0	1	ND	0	ND	ND	1	0	3	ND	ND	1	3	ND	0	ND	ND	1	ND	ND
2016	Hamadan	19	Animal	Disk diffusion	10	8	12	ND	ND	ND	4	1	5	13	ND	2	7	ND	0	ND	12	1	ND	ND
2013-2014	Hamadan	3	Human	Disk diffusion	ND	ND	ND	ND	ND	ND	ND	0	2	ND	ND	3	2	ND	1	2	ND	1	ND	ND
2014-2015	Isfahan	6	Animal	Disk diffusion	0	0	ND	0	ND	ND	0	0	3	ND	ND	0	0	ND	0	ND	ND	2	ND	ND
2006–2008	Isfahan	4	Animal	Disk diffusion	0	1	ND	2	ND	ND	0	0	2	ND	ND	0	1	ND	0	ND	ND	1	ND	ND
2011-2012	Isfahan-Shahrekord	3	Animal	Disk diffusion	0	0	ND	1	ND	ND	0	0	1	ND	ND	1	1	ND	0	ND	ND	1	ND	ND
2008-2009	Isfahan-Yazd	8	Animal	Disk diffusion	0	0	ND	1	ND	ND	0	0	6	ND	ND	0	2	ND	0	ND	ND	2	ND	ND
2015-2016	Kurdistan	8	Animal	Disk diffusion agar dilution	5	6	ND	ND	ND	ND	ND	ND	7	ND	ND	0	6	ND	0	0	ND	ND	ND	ND
2013	Mashhad	27	Animal	Disk diffusion	3	6	ND	4	ND	5	ND	3	17	ND	ND	9	12	ND	1	ND	ND	13	ND	ND
2012	Mashhad	27	Animal	Disk diffusion	2	1	9	7	1	0	2	1	13	ND	ND	0	15	ND	0	ND	ND	18	ND	ND
2014-2015	Mazandaran-Golestan	41	Animal	Disk diffusion	17	15	ND	16	ND	ND	15	ND	36	ND	ND	2	36	ND	2	ND	ND	32	ND	ND
2010	Rafsanjan	12	Animal	Disk diffusion	ND	ND	ND	ND	ND	ND	ND	ND	ND	12	ND	0	0	ND	12	ND	ND	0	ND	ND
2014	Shahrekord	13	Animal	Disk diffusion	0	3	ND	4	ND	ND	0	0	7	ND	ND	0	5	ND	0	ND	ND	4	ND	ND
2009-2010	Shahrekord	15	Animal	Disk diffusion	2	0	ND	2	ND	ND	1	0	7	ND	ND	0	4	ND	0	ND	ND	5	ND	ND
ND	Shahrekord	4	Animal	Disk diffusion	0	0	ND	1	ND	ND	0	0	2	ND	ND	0	0	ND	0	ND	ND	2	ND	ND
2011–2013	Shiraz	17	Animal	Disk diffusion	5	ND	0	3	ND	0	ND	1	2	ND	4	5	14	ND	1	ND	10	8	ND	ND
ND	Shiraz	10	Animal	Disk diffusion E-test	1	ND	ND	ND	ND	ND	ND	2	1	1	6	1	0	ND	1	ND	ND	ND	ND	4
ND	Shiraz	10	Animal	Disk diffusion *E*-test	1	ND	ND	ND	ND	ND	ND	2	1	ND	6	1	0	ND	1	ND	ND	ND	ND	4
2011-2012	Tehran	28	Animal	Disk diffusion	1	5	4	ND	2	2	1	1	6	ND	ND	1	13	ND	1	ND	ND	24	ND	ND
2012	Tehran	39	Animal	Disk diffusion	32	30	ND	ND	ND	ND	35	27	38	31	ND	33	34	ND	0	ND	ND	36	ND	ND
2010	Tehran	33	Animal	Disk diffusion	25	ND	ND	ND	ND	0	ND	ND	31	ND	ND	ND	10	ND	ND	ND	ND	0	ND	ND
2008–2010	Tehran	23	Animal	Disk diffusion	1	3	8	ND	ND	1	1	0	15	ND	ND	1	15	ND	0	ND	ND	18	ND	ND
2008-2009	Tehran	12	Human	Disk diffusion	2	ND	2	ND	ND	1	1	1	3	ND	6	1	6	0	0	ND	ND	6	5	ND
2006-2007	Tehran	22	Animal	Disk diffusion	0	3	2	ND	1	1	0	0	4	ND	ND	0	9	ND	0	ND	ND	18	ND	ND
2004-2005	Tehran	5	Human	Disk diffusion	1	ND	0	ND	ND	0	0	0	0	ND	2	0	4	0	0	ND	5	5	4	5
ND	Tonekabon	8	Animal	Disk diffusion *E*-test	8	ND	ND	ND	ND	ND	ND	2	1	ND	7	2	0	ND	1	ND	ND	ND	ND	8
ND	Tonekabon	9	Animal	Disk diffusion	9	9	ND	ND	ND	ND	1	2	1	ND	ND	1	0	ND	0	ND	ND	ND	ND	9

Abbreviations: AMP: ampicillin; AMX: amoxicillin; CST: colistin; NFX: enrofloxacin; SPT: spectinomycin; NEO: neomycin; STR: streptomycin; CHL: chloramphenicol; TET: tetracycline; TMP/SMX: trimethoprim/sulfamethoxazole; CTX: cefotaxime; ERY: erythromycin; CIP: ciprofloxacin; IPM: imipenem; GEN: gentamicin; MEM: meropenem; CEF: cephalothin; NAL: nalidixic acid; CAZ: ceftazidime; LEX: cephalexin; AST: antimicrobial susceptibility testing; ND: not determined.

**Table 3:** Antibiotic resistance profiles of *C. lari*.

Year	City	Strains (*n*)	Origin	AST	Antibiotic resistance (*n*)																			
					AMP	AMX	CST	NFX	SPT	NEO	STR	CHL	TET	TMP-SMX	CTX	ERY	CIP	IPM	GEN	MEM	CEF	NAL	CAZ	LEX
ND	Shiraz	12	Animal	Disk diffusion *E*-test	3	ND	ND	ND	ND	ND	ND	2	1	2	8	1	0	ND	1	ND	ND	ND	ND	5
ND	Shiraz	12	ND	Disk diffusion *E*-test	3	ND	ND	ND	ND	ND	ND	2	1	ND	8	1	0	ND	1	ND	ND	ND	ND	5
ND	Tonekabon	8	ND	Disk diffusion *E*-test	8	ND	ND	ND	ND	ND	ND	2	1	ND	8	0	0	ND	2	ND	ND	ND	ND	7
ND	Tonekabon	7	Animal	Disk diffusion	7	7	ND	ND	ND	ND	1	2	1	ND	ND	0	0	ND	0	ND	ND	ND	ND	7

## Data Availability

There are no original data associated with this review.
